# A three-gene expression score for predicting clinical benefit to anti-PD-1 blockade in advanced renal cell carcinoma

**DOI:** 10.3389/fimmu.2024.1374728

**Published:** 2024-04-10

**Authors:** Yoel Z. Betancor, Miriam Ferreiro-Pantín, Urbano Anido-Herranz, Mar Fuentes-Losada, Luis León-Mateos, Silvia Margarita García-Acuña, Vanessa Vaamonde-Rodríguez, Beatriz García-Pinel, Víctor Cebey-López, Rosa Villaverde-Viaño, Helena Lombardía-Rodríguez, Martin Kotrulev, Natalia Fernández-Díaz, Iria Gomez-Tourino, Carlos Fernández-Baltar, Jorge García-González, Jose M. C. Tubio, Rafael López-López, Juan Ruiz-Bañobre

**Affiliations:** ^1^ Translational Medical Oncology Group (ONCOMET), Health Research Institute of Santiago de Compostela (IDIS), University Clinical Hospital of Santiago de Compostela, University of Santiago de Compostela (USC), Santiago de Compostela, Spain; ^2^ Centre for Research in Molecular Medicine and Chronic Diseases (CiMUS), University of Santiago de Compostela (USC), Santiago de Compostela, Spain; ^3^ Department of Medical Oncology, University Clinical Hospital of Santiago de Compostela (SERGAS), University of Santiago de Compostela (USC), Santiago de Compostela, Spain; ^4^ Centro de Investigación Biomédica en Red de Cáncer (CIBERONC), Instituto de Salud Carlos III, Madrid, Spain; ^5^ Department of Pathology, University Clinical Hospital of Santiago de Compostela, University of Santiago de Compostela (USC), Santiago de Compostela, Spain; ^6^ Health Research Institute of Santiago de Compostela (IDIS), Santiago de Compostela, Spain; ^7^ Department of Urology, Complexo Hospitalario Universitario de Pontevedra, Pontevedra, Spain

**Keywords:** nivolumab, biomarker, gene expression, immunotherapy, kidney cancer

## Abstract

In the advanced renal cell carcinoma (RCC) scenario, there are no consistent biomarkers to predict the clinical benefit patients derived from immune checkpoint blockade (ICB). Taking this into consideration, herein, we conducted a retrospective study in order to develop and validate a gene expression score for predicting clinical benefit to the anti-PD-1 antibody nivolumab in the context of patients diagnosed with advanced clear cell RCC enrolled in the CheckMate-009, CheckMate-010, and CheckMate-025 clinical trials. First, a three-gene expression score (3GES) with prognostic value for overall survival integrating HMGA1, NUP62, and ARHGAP42 transcripts was developed in a cohort of patients treated with nivolumab. Its prognostic value was then validated in the TCGA-KIRC cohort. Second, the predictive value for nivolumab was confirmed in a set of patients from the CheckMate-025 phase 3 clinical trial. Lastly, we explored the correlation of our 3GES with different clinical, molecular, and immune tumor characteristics. If the results of this study are definitively validated in other retrospective and large-scale, prospective studies, the 3GES will represent a valuable tool for guiding the design of ICB-based clinical trials in the aRCC scenario in the near future.

## Introduction

The emergence of immunotherapy, particularly programmed cell death-1 (PD-1) antibodies, has revolutionized the management of patients diagnosed with clear cell renal cell carcinoma (ccRCC) over the last decade. Since the approval of nivolumab (an anti-PD-1 antibody) in November 2015 for the treatment of patients with advanced ccRCC (accRCC) who received previous antiangiogenic therapy, new immunotherapy-based combination regimens have been approved for previously untreated patients (1). Today, in the first-line setting, there are several immune checkpoint blockade (ICB)-based alternatives that have been approved by the main regulatory agencies after demonstrating significant overall survival improvements in randomized, phase 3 clinical trials: pembrolizumab (an anti-PD-1 antibody) plus either axitinib or lenvatinib (both tyrosine kinase inhibitors) (2, 3), nivolumab plus cabozantinib (a tyrosine kinase inhibitor) (4), and nivolumab plus ipilimumab (an anti-CTLA-4 antibody) (this combination only for those patients with intermediate- or poor-risk aRCC) (5). In the second and subsequent lines of therapy, there are different available single-agent options beyond nivolumab such as tyrosine kinase and mTOR inhibitors (5). Furthermore, very recently, pembrolizumab has been approved for the treatment of adults with ccRCC at increased risk of recurrence following nephrectomy or following nephrectomy and resection of metastatic lesions (6).

In parallel with the growth of the immunotherapeutic armamentarium, to identify ccrRCC patients most likely to benefit from ICB has become a priority. Over the last years, a plethora of studies have evaluated the role of different prognostic and/or predictive biomarkers for ICB in accRCC. Numerous translational research initiatives have explored the role of different molecular markers such as PD-L1 (1, 7, 8), tumor mutational burden (TMB) (9–12), PBRM1 loss-of-function mutations (13), alterations in DNA damage response and repair genes (13), gene expression signatures (13), and T-cell receptor clonality in the tumor microenvironment (14). Other host-related biomarkers such as obesity (13, 15), presence of pancreatic metastases (16), the International Metastatic RCC Database Consortium (IMDC) risk score (7), or the gut microbiome have also been evaluated. Nevertheless, to date, the IMDC risk score is the only biomarker used in clinical practice as a selection criterion to treat patients with the combination regimen of nivolumab plus ipilimumab (7).

Taking this into consideration, herein, we conducted a retrospective study in order to develop and validate a gene expression score for predicting clinical benefit to the anti-PD-1 antibody nivolumab in the context of patients diagnosed with accRCC enrolled in the CheckMate-009, CheckMate-010, and CheckMate-025 clinical trials. Additionally, we explored the correlation of our three-gene expression score (3GES) with different clinical, molecular, and immune tumor characteristics.

## Patients and methods

### Study design and patient population

The design and primary outcomes of the CheckMate-009, CheckMate-010, and CheckMate-025 trials were described in previous reports (1, 17, 18). Briefly, CheckMate-009 (17) was an open-label, parallel, four-group, phase 1 trial that investigated the pharmacodynamic immunomodulatory activity, efficacy, and safety of nivolumab in patients with previously treated accRCC; CheckMate-010 was a blinded, randomized, multicenter phase 2 trial that evaluated the dose–response relationship, efficacy, and safety of nivolumab in patients with previously treated accRCC (18); and CheckMate-025 was a two-arm, randomized, open-label, phase 3 study that compared nivolumab with everolimus in patients with previously treated accRCC (1). This is a *post*-*hoc* pooled analysis of 311 patients with available clinical, molecular, and immune tumor data from the CheckMate-009, CheckMate-010, and CheckMate-025 trials (16, 45, and 250 patients, respectively) (1, 17, 18). For the purpose of our analyses, our efficacy endpoints were overall survival (OS), disease control rate (DCR), and overall response rate (ORR). Tumor responses were assessed according to the Response Evaluation Criteria in Solid Tumors guidelines version 1.1. Additionally, the TCGA Kidney Renal Clear Cell Carcinoma (TCGA-KIRC) cohort was used as an external validation set.

All clinical and molecular tumor data (generated from pretreatment tumor samples) used for this retrospective study have been made freely available through a **Supplementary Material** by Braun et al. (9) Briefly, RNA-seq data from the CheckMate-010 and CheckMate-025 cohorts were aligned using STAR (19), quantified using RSEM (20), and evaluated for quality using RNA-seQC2 (21). Samples were excluded if they had an interquartile range of log_2_[transcript per million (TPM) + 1] < 0.5 (indicating low dynamic range), had less than 15,000 genes detected (indicating low library complexity), had an End 2 Sense Rate < 0.90, or End 1 Sense Rate > 0.10 (as defined by RNA-seqQC2, indicating strand bias). For samples where RNA-seq was performed in duplicates, the run with a higher interquartile range of log_2_(TPM + 1), used as a surrogate for better quality data, was used. For the CheckMate-009 cohort, the previously published TPM matrix was used (22). Genes that were not expressed in any of the samples (in each cohort independently) then upper quartile-normalized the TPMs to an upper quartile of 1,000 and log_2_-transformed them were filtered. Since the sequencing had been performed in four separate batches, principal component analysis (PCA) was used to evaluate for batch effects, and four batches were observed. These four batches were corrected by using ComBat (23). Subsequently, a PCA was performed on the ComBat-corrected expression matrix to confirm that batch effects had been adequately corrected. Moreover, a constant that was equal to the first integer above the minimum negative expression value obtained post-ComBat (constant of +21) was used to eliminate negative gene expression values that were a by-product of ComBat correction. The ComBat-corrected expression matrix was used for all downstream analyses. RNA-seq data from the TCGA-KIRC were downloaded from the UCSC Xena Browser (dataset identification: TCGA.KIRC.sampleMap/HiSeqV2) as log_2_(normalized_count+1) and transformed to TPM values. The expression levels of all the genes were independently dichotomized for each cohort into high and low using the maximally selected rank statistic maxstat.test() function from *maxstat* R package. Computational immune cell deconvolution was carried out with EPIC version 1.1.7 R package (24).

Statements confirming compliance with ethical regulations, the committees that approved the protocol of CheckMate studies, and confirmation of informed consent from all study participants are included in the previous publications describing these trials (NCT01358721, NCT01354431, and NCT01668784) (1, 17, 18).

### Statistical analysis

Survival estimates were calculated by the Kaplan–Meier method, and groups were compared with the log-rank test. The Cox proportional hazards regression model was used to evaluate factors independently associated with OS. Baseline clinicopathological variables included in the multivariable analysis were selected according to statistical significance in univariable analysis (cutoff, *P* < 0.05) (**Supplementary Table 1**). The proportional hazard assumption was verified with the Schoenfeld residual method. Factors associated with disease control (DC) and response were tested with logistic regression in univariable analyses. Variables included in the final multivariable model were selected according to their statistical significance in univariable analysis (cutoff, *P* < 0.05). Time-dependent ROC curves were used as a complementary method to assess the discriminative capacity of 3GES for OS. Biomarker–treatment interaction was evaluated with the likelihood ratio test. Comparisons between patient and disease characteristics were carried out using chi-squared or Fisher exact tests. Comparisons between estimated cell fractions and immune exhaustion marker expression levels were carried out using the Wilcoxon rank-sum test. All *P-*values were two-sided, and those less than 0.05 were considered statistically significant. The Bonferroni and the Benjamini–Hochberg (B-H) procedures were used to control the family-wise error rate (FWER) and the false discovery rate (FDR), respectively, in the case of multiple comparisons. All statistical analyses were performed using R version 4.2.2 (Vienna, Austria).

## Results

### Development and validation of a novel three-gene expression score with prognostic significance

**Figure 1:**
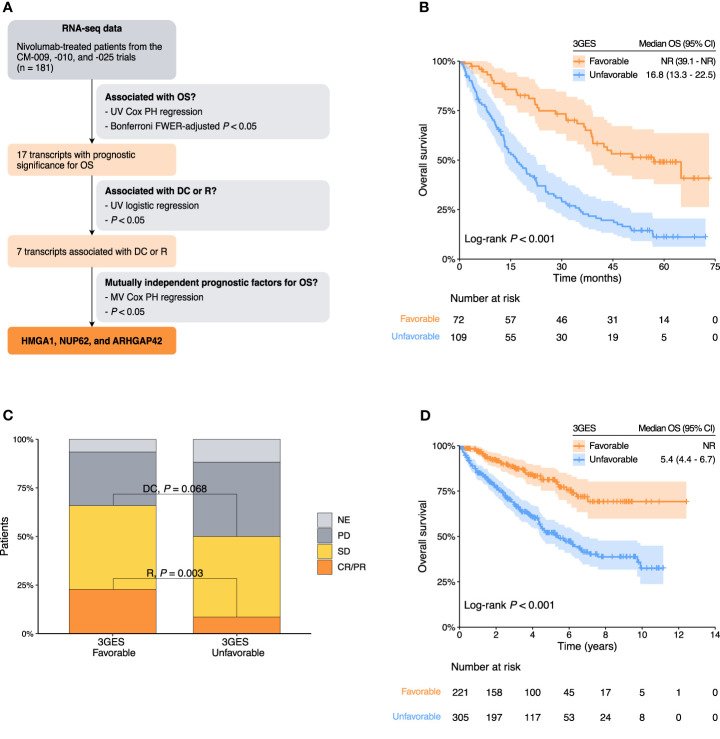
**(A)** Flow diagram of the selection process of final transcripts integrated into the three-gene expression score (3GES). **(B)** Kaplan–Meier overall survival estimates of nivolumab-treated patients from the pooled cohort of the CheckMate-009, CheckMate-010, and CheckMate-025 trials according to the 3GES. **(C)** Nivolumab response distribution by 3GES. **(D)** Kaplan–Meier overall survival estimates of patients from the TCGA-KIRC cohort. Abbreviations: 3GES, three-gene expression score; CI, confidence interval; CM, CheckMate; CR, complete response; DC, disease control; FWER, family-wise error rate; MV, multivariable; NE, not evaluable; NR, not reached; OS, overall survival; PD, progressive disease; PH, proportional hazard; PR, partial response; R, response; SD, stable; UV, univariable.

First, to identify genes with prognostic significance among ccRCC patients treated with immunotherapy, we evaluated the association of 43,893 transcripts with OS by using univariable Cox proportional hazard models in a pooled cohort of 181 patients treated with nivolumab from the CheckMate-009, CheckMate-010, and CheckMate-025 trials. Seventeen out of the 43,893 transcripts evaluated were significantly associated with OS (Bonferroni FWER-adjusted *P* < 0.05) (**Figure 1A**, **Supplementary Table 2**). Next, to further optimize the selection of genes, we filtered out those transcripts that exhibited no correlation with either disease control or response. Seven out of 17 genes showed a statistically significant association with either disease control, response, or both (**Figure 1A**, **Supplementary Table 3**). Importantly, the direction of this association for each gene was clinically consistent with that previously identified with OS. Lastly, we used a multivariable Cox proportional hazard model to obtain a final panel of three independent prognostic genes (**Figure 1A**, **Supplementary Table 4**). A high expression of HMGA1 and NUP62 was associated with a worse OS (HR = 1.60, 95% CI 1.05–2.46, *P* = 0.031 and HR = 1.74, 95% CI 1.17–2.60, *P* = 0.007, respectively) (**Supplementary Table 4**). On the contrary, a low expression of ARHGAP42 was associated with a worse OS (HR = 1.74, 95% CI 1.15–2.63, *P* = 0.008) (**Supplementary Table 4**).

Considering the amount of these three adverse prognostic genes (high HMGA1 = 1 point, high NUP62 = 1 point, and low ARHGAP42 = 1 point), we developed a 3GES to segregate patients into two risk categories. Patients without any adverse prognostic gene (0 points) were classified in the favorable-risk category [40%, *n* = 72; median OS = not reached (NR) (95% CI, 39.1–NR)], and patients with one or more adverse prognostic genes (1 to 3 points) were classified in the unfavorable-risk category [60%, *n* = 109; median OS = 16.8 months (95% CI, 13.3–22.5)]. The Kaplan–Meier curves depicting these two risk categories are presented in **Figure 1B**. Favorable 3GES risk category was significantly associated with a better OS in univariable (HR = 0.32, 95% CI 0.21–0.48, *P* < 0.001; CheckMate-009 HR = 0.22, *P* = 0.182; CheckMate-009 HR = 0.32, *P* = 0.011; CheckMate-009 HR = 0.32, *P* < 0.001) and multivariable (HR = 0.36, 95% CI 0.24–0.56, *P* < 0.001) analyses (**Table 1**). These results were supported by a complementary time-dependent ROC curve analysis (**Supplementary Figure 1A**). DCR and ORR were higher among favorable 3GES risk category patients (DCR for favorable vs. unfavorable 3GES risk category patients: 65% vs. 51%, *P* = 0.068; ORR for favorable vs. unfavorable 3GES risk category patients: 33% vs. 14%, *P* = 0.003; **Figure 1C**). Moreover, favorable 3GES risk category patients presented a higher probability of disease control and response in univariable [DC: odds ratio (OR) = 1.78, 95% CI 0.96–3.29, *P* = 0.066; response: OR = 3.13, 95% CI 1.51–6.54, *P* = 0.002] and multivariable (DC: OR = 1.85, 95% CI 0.93–3.70, *P* = 0.081; response: OR = 3.12, 95% CI 1.39–6.98, *P* = 0.006) analyses (**Table 1**). Additionally, we compare the prognostic significance of the 3GES with other well-recognized gene expression signatures such as the angiogenesis score, the T effector cell infiltration score, the myeloid cell infiltration score, the JAVELIN immune infiltration score, and the tumor inflammation score. Again, the 3GES retained its independent prognostic value as shown in **Supplementary Table 5**.

**Table 1:** Univariable and multivariable Cox regression analyses for overall survival and logistic regression analyses for disease control and response among nivolumab-treated patients included in the pooled cohort of the CheckMate-009, CheckMate-010, and CheckMate-025 trials.

	Univariable analysis		Multivariable analysis	
Characteristics	HR (95% CI)	*P*	HR (95% CI)	*P*
Overall survival				
3GES (favorable vs. unfavorable)	0.32 (0.21–0.48)	<0.001	0.36 (0.24–0.56)	<0.001
Sex (male vs. female)	1.69 (1.09–2.63)	0.020	1.40 (0.88–2.24)	0.156
MSKCC risk (favorable vs. intermediate/poor)	0.44 (0.29–0.68)	<0.001	0.48 (0.31–0.75)	0.001
Sarcomatoid or rhabdoid differentiation (yes vs. no)	1.73 (1.03–2.89)	0.039	1.49 (0.88–2.51)	0.134
	OR (95% CI)	*P*	OR (95% CI)	*P*
Disease control				
3GES (favorable vs. unfavorable)	1.78 (0.96–3.29)	0.066	1.85 (0.93–3.70)	0.081
Sex (male vs. female)	0.89 (0.45–1.77)	0.737	1.17 (0.53–2.56)	0.699
MSKCC risk (favorable vs. intermediate/poor)	3.05 (1.47–6.32)	0.003	2.67 (1.26–5.66)	0.011
Sarcomatoid or rhabdoid differentiation (yes vs. no)	0.36 (0.14–0.97)	0.043	0.43 (0.15–1.19)	0.103
Response				
3GES (favorable vs. unfavorable)	3.13 (1.51–6.54)	0.002	3.12 (1.39–6.98)	0.006
Sex (male vs. female)	0.56 (0.26–1.21)	0.141	0.59 (0.25–1.43)	0.248
MSKCC risk (favorable vs. intermediate/poor)	1.11 (0.51–2.44)	0.791	0.91 (0.39–2.16)	0.835
Sarcomatoid and/or rhabdoid differentiation (yes vs. no)	0.95 (0.29–3.05)	0.929	1.12 (0.33–3.78)	0.859

Twenty-two cases lacking sarcomatoid and/or rhabdoid differentiation data were removed from these analyses (number of cases included, n = 159).

3GES, three-gene expression score; CI, confidence interval; HR, hazard ratio; OR, odds ratio; MSKCC, Memorial Sloan Kettering Cancer Center.

Once the 3GES was developed, we went to validate its prognostic significance in an independent dataset, the TCGA-KIRC cohort. The Kaplan–Meier curves depicting these two risk categories are presented in **Figure 1D**. Consistent with our previous findings, favorable 3GES risk category was significantly associated with a better OS in univariable (HR = 0.35, 95% CI 0.24–0.50, *P* < 0.001) and multivariable (HR = 0.33, 95% CI 0.22–0.50, *P* < 0.001) analyses (**Table 2**). These results were supported by a complementary time-dependent ROC analysis (**Supplementary Figure 1B**).

**Table 2:** Univariable and multivariable Cox regression analyses for overall survival among patients from the TCGA-KIRC cohort.

	Univariable analysis		Multivariable analysis	
Characteristics	HR (95% CI)	*P*	HR (95% CI)	*P*
3GES (favorable vs. unfavorable)	0.35 (0.24–0.50)	<0.001	0.33 (0.22–0.50)	<0.001
AJCC stage at diagnosis (III–IV vs. I–II)	3.95 (2.87–5.45)	<0.001	3.73 (2.57–5.42)	<0.001
Hemoglobin (low vs. normal/high)	2.15 (1.52–3.06)	<0.001	1.36 (0.93–2.00)	0.116
Calcium (high vs. normal/low)	4.36 (2.20, 8.62)	<0.001	2.28 (1.14–4.55)	0.020

Three cases lacking AJCC stage at diagnosis data, 80 cases lacking hemoglobin data, and 164 cases lacking calcium data were removed from these analyses (number of cases included, n = 355).

3GES, three-gene expression score; CI, confidence interval; HR, hazard ratio.

### Evaluation of the tumor-subtype specificity of the three-gene expression score

Next, to assess the tumor-subtype specificity of the 3GES, we proceeded to validate its performance for OS prognostication using two independent clinical datasets representing distinct RCC subtypes: TCGA-Kidney Renal Papillary Cell Carcinoma (TCGA-KIRP) and TCGA-Kidney Chromophobe (TCGA-KICH). In the TCGA-KIRP cohort, the three genes consistently demonstrated independent prognostic significance, aligning with our previous analyses in ccRCC (HMGA1: HR = 2.71, 95% CI 1.37–5.38, *P* = 0.004; NUP62: HR = 2.38, 95% CI 1.18–4.81, *P* = 0.015; ARHGAP42: HR = 4.82, 95% CI 1.47–15.81, *P* = 0.009) (**Supplementary Table 6**). However, when attempting to evaluate the 3GES in this cohort, the Cox model did not converge due to the low prevalence of one of the two binary predictors (7 favorable vs. 279 unfavorable cases). In contrast, in the TCGA-KICH cohort, none of the three genes exhibited independent prognostic significance. While HMGA1 and NUP62 showed a trend consistent with our previous analyses in ccRCC, the association of ARHGAP42 with OS was in the opposite direction (**Supplementary Table 6**). Considering these findings, the prognostic significance of the 3GES was not further evaluated in this particular cohort.

### Evaluation of the predictive value of the three-gene expression score

**Figure 2:**
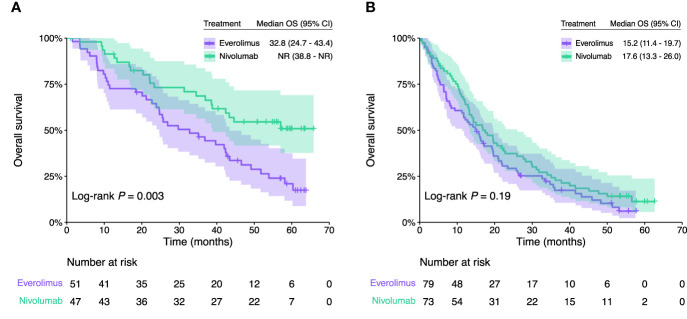
Kaplan–Meier overall survival estimates of patients from the CheckMate-025 trial according to the treatment arm (nivolumab vs. everolimus) in **(A)** favorable and **(B)** unfavorable 3GES risk groups. CI, confidence interval; OS, overall survival; NR, not reached.

To further explore the predictive value of our 3GES when patients are treated with immunotherapy, we specifically interrogated those patients with available RNA sequencing and clinical data from the CheckMate-025 study (*n* = 250). Among favorable 3GES risk category patients, nivolumab monotherapy significantly improved the OS compared with everolimus (nivolumab arm, median OS = NR, 95% CI 38.8–NR vs. everolimus arm, mOS = 32.8, 95% CI 24.7–43.4), with a reduction of death risk of 54% (HR = 0.46, 95% CI 0.27–0.79, *P* = 0.003) (**Figure 2A**). Conversely, among unfavorable 3GES risk category patients, there were no significant differences in terms of OS based on the allocated treatment arm (nivolumab arm, median OS = 17.6, 95% CI 13.3–26.0 vs. everolimus arm, mOS = 15.2, 95% CI 11.4–19.7; HR = 0.79, 95% CI 0.56–1.12, *P* = 0.19) (**Figure 2B**). Importantly, the 3GES–treatment interaction was statistically significant whether unadjusted (LRT *P* < 0.001, concordance = 0.64) or after adjustment for previously confirmed independent prognostic factors (sex, MSKCC risk group, and presence of sarcomatoid and/or rhabdoid histological differentiation) (LRT *P* < 0.001, concordance = 0.68).

### Clinical, molecular, and immune correlates of the three-gene expression score

To fully characterize our 3GES, we evaluated its correlation with available baseline patient and disease characteristics among the 311 subjects included in the CheckMate-009, CheckMate-010, and CheckMate-025 trial pooled cohorts (**Supplementary Table 7**). Forty percent (*n* = 123) of the patients had a favorable 3GES risk, while 60% (*n* = 188) had an unfavorable 3GES risk; 5% (*n* = 16) of the patients come from CheckMate-009, 15% (*n* = 45) from CheckMate-010, and 80% (*n* = 250) from CheckMate-025. The distribution of different patient and disease characteristics according to the 3GES is shown in **Supplementary Table 7**. Notably, in the favorable 3GES risk group, there were a higher proportion of patients with favorable MSKCC risk (*P* = 0.039), with absence of sarcomatoid and/or rhabdoid histological differentiation (*P* = 0.042), and with a lower copy number alteration burden [as measured by the weighted genome integrity index (wGII)] (*P* = 0.035). Importantly, there were no statistically significant differences in the cohort of origin (CheckMate-025 trial vs. CheckMate-009 and CheckMate-010 trials) among favorable and unfavorable 3GES risk cases.

Next, we evaluated whether any individual mutation or copy number alteration was associated with the 3GES. Interestingly, the tumors of patients from the favorable 3GES risk group presented a significant enrichment in *PBRM1* loss-of-function mutations (B-H FDR-adjusted *P* = 0.010) and the amplification 8Q24.3 (B-H FDR-adjusted *P* = 0.017). There were no statistically significant differences in other molecular alterations among favorable and unfavorable 3GES risk cases.

**Figure 3:**
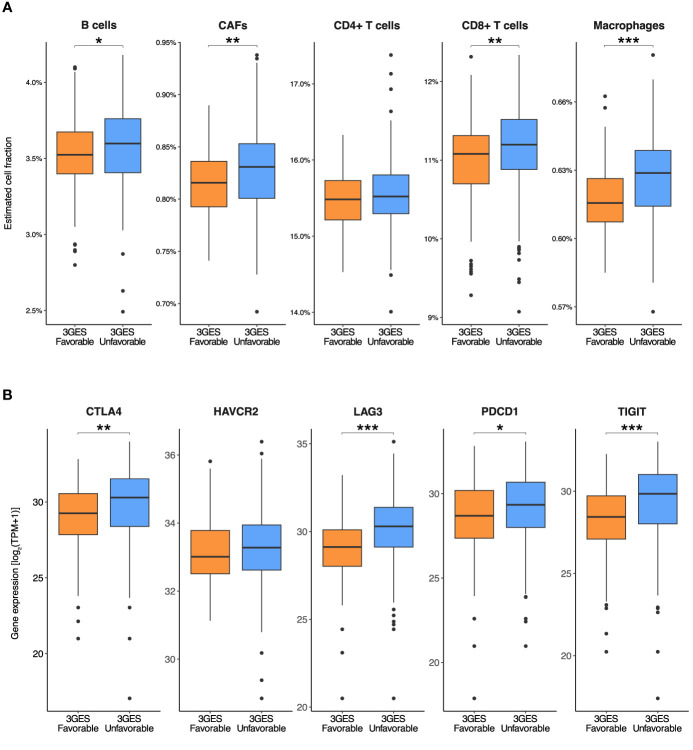
Comparisons between **(A)** estimated cell fractions and **(B)** immune exhaustion marker expression levels by 3GES among patients from the pooled cohort of the CheckMate-009, CheckMate-010, and CheckMate -025 trials. In the boxplots, whiskers represent the variability outside the upper and lower quartiles, the middle line in the box stands for median, the bounds of the box stand for upper and lower quartiles, and the dots stand for outliers. *P-*values of Wilcoxon rank-sum tests within pairs of gene groups are indicated by asterisks (*P < 0.05, **P < 0.01, ***P < 0.001). 3GES, three-gene expression score; CAFs, cancer-associated fibroblasts; TPM, transcripts per million.

Lastly, we evaluated the tumor microenvironment through computational immune cell deconvolution. Surprisingly, the tumors of patients from the unfavorable 3GES risk category were infiltrated by a significantly higher proportion of immune cells such as B cells (B-H FDR-adjusted *P* = 0.036), CD8^+^ T cells (B-H FDR-adjusted *P* = 0.004), and macrophages (B-H FDR-adjusted *P* < 0.001) (**Figure 3A**). The estimated proportion of NK cells was negligible for all samples. To further explore this, we examined the level of expression of different immune exhaustion markers on these tumor samples. Compared with tumors from patients of the favorable 3GES risk category, those from the unfavorable-risk group presented a significantly higher expression of immune exhaustion markers such as *CTLA4* (B-H FDR-adjusted *P* = 0.001), *LAG3* (B-H FDR-adjusted *P* < 0.001), *PDCD1* (B-H FDR-adjusted *P* = 0.023), and *TIGIT* (B-H FDR-adjusted *P* < 0.001) (**Figure 3B**). Consistently with this finding and with the clinical value of our 3GES, those tumors from the unfavorable-risk group exhibit a significantly higher proportion of cancer-associated fibroblasts (CAFs) (B-H FDR-adjusted *P* = 0.003) (**Figure 3B**).

## Discussion

Nowadays, in the accRCC scenario, there are no consistent ICB predictive biomarkers. Even though multiple studies have been conducted to discover predictive biomarkers in this context, only the IMDC risk score is used in clinical practice to select those patients who are candidates for treatment with the combination regimen nivolumab plus ipilimumab (7). Moreover, although microsatellite instability and TMB are also FDA-approved as predictive biomarkers for pembrolizumab in a tissue/site-agnostic cancer indication, their utility in ccRCC is anecdotal and debatable. Taking this into consideration, herein, we conducted a retrospective study in order to develop and validate a gene expression score for predicting clinical benefit to the anti-PD-1 antibody nivolumab in the context of patients diagnosed with accRCC enrolled in the CheckMate-009, CheckMate-010, and CheckMate-025 clinical trials. Additionally, we explored the correlation of our 3GES with different clinical, molecular, and immune tumor characteristics.

First, we systematically developed a novel 3GES with prognostic value in a pooled cohort of 181 patients treated with nivolumab from the CheckMate-009, CheckMate-010, and CheckMate-025 trials. Considering the expression level of three independent prognostic genes, we developed a simple model to segregate patients into two risk categories based on the hazard of death (favorable and unfavorable prognostic groups), which importantly was also associated with DC and response. Next, we evaluated and validated the prognostic significance of our score in an independent dataset, the TCGA-KIRC cohort. Moreover, to assess the tumor-subtype specificity of the 3GES, we evaluated its performance for OS prognostication in two independent clinical datasets representing distinct RCC subtypes: TCGA-KIRP and TCGA-KICH. In both cohorts, 3GES was not able to demonstrate any prognostic value, confirming a potential tumor-subtype specificity for ccRCC.

Regarding the genes included in the risk score, none of them have been previously described as either a prognostic or predictive biomarker in RCC. However, *in-vitro* functional analysis in human RCC cell lines specifically revealed that HMGA1 knockdown markedly inhibited colony formation, significantly induced apoptosis, inhibited invasion potential, and induced anoikis, suggesting this molecule as a potential target for novel therapeutic modalities for advanced RCC (25). Though no relevant information is available related to the functional role in RCC of the other evaluated transcripts, there are some limited data on other tumor types. For example, in head and neck cancer, NUP62 plays a role in stabilizing NUP88, which ultimately leads to the activation of the NF-κB pathway, promoting the proliferation of cancer cells (26). In nasopharyngeal carcinoma, elevated expression of ARHGAP42 is associated with reduced metastasis-free survival. This association is supported by *in-vitro* data in nasopharyngeal carcinoma cell lines, demonstrating that ARHGAP42 promotes migration capacity and invasiveness of tumor cells (27). However, in ccRCC, we found the opposite effect: a low expression of ARHGAP42 was associated with a worse OS.

Second, once the prognostic value of our 3GES was confirmed, we further evaluated its capacity as a predictive biomarker for anti-PD-1 blockade. For this purpose, we specifically interrogated our score among patients enrolled in the CheckMate-025 study, a two-arm, randomized, open-label, phase 3 study of nivolumab in comparison with everolimus. The 3GES showed a statistically significant interaction with the treatment arm. Among patients with an unfavorable 3GES risk, there was no significant difference in terms of OS based on the allocated treatment arm, while among those with a favorable score, nivolumab monotherapy significantly improved the OS compared with everolimus.

Lastly, according to clinical and pathological features, we found an enrichment of different characteristics classically associated with better prognosis among those cases with a favorable 3GES risk such as a favorable MSKCC risk score (28) and the absence of sarcomatoid and/or rhabdoid histological differentiation (29, 30). Moreover, when we evaluated the molecular profile of tumors, we found a higher proportion of PBRM1 loss-of-function mutations and a lower proportion of the amplification 8Q24.3 among those cases with a favorable 3GES risk. Although initially there was evidence supporting the role of *PBRM1* loss-of-function mutations as a positive predictive biomarker for the anti-PD-1 antibody nivolumab (9, 22, 31), new data claiming the opposite role (32) has hampered its translation to the clinic. Interestingly, regarding the amplification of 8Q24.3, Braun et al. reported a higher frequency of this molecular alteration among immune-infiltrated ccRCC tumors (9). Additionally, favorable 3GES risk cases presented a lower wGII, a genomic characteristic previously associated with a less aggressive phenotype compared with those ccRCC tumors with a higher wGII (33). From an immune perspective, when we evaluated the tumor microenvironment through computational immune cell deconvolution, we found that those tumors from patients with a favorable 3GES risk overall presented lower immune cell infiltrates compared with their counterparts with an unfavorable 3GES risk. However, despite having higher levels of immune cell infiltration, unfavorable 3GES risk tumors presented a higher expression of immune exhaustion markers, which could explain their worse clinical outcome. Furthermore, patients from the unfavorable 3GES risk group exhibited a higher proportion of CAFs, a fibroblast population with a well-documented immunosuppressive role that limits anti-PD-1 blockade efficacy (34, 35). Based on this, one could hypothesize that patients with unfavorable 3GES risk tumors would benefit from combination strategies against CTLA-4 and other non-classical immune checkpoint molecules such as LAG-3 or TIGIT. Moreover, due to the higher proportion of CAFs, emerging therapies targeting this cellular population by either depleting them, reducing their tumor-promoting and immunosuppressive functions, or even by reprogramming them to a more quiescent state (36) are potential strategies to improve clinical outcomes in this population characterized by an unfavorable 3GES. On the other hand, patients with a favorable 3GES could be ideal candidates for less toxic therapeutic strategies involving anti-PD-1 monotherapy. In this selected population, this approach would potentially replace the current first-line combination strategies, which include nivolumab plus ipilimumab, nivolumab plus cabozantinib, or pembrolizumab plus axitinib or lenvatinib. Moreover, to conduct prospective clinical trials to test de-escalation therapeutic strategies in favorable 3GES ccRCC patients, the first and feasible step would be to retrospectively assess the predictive role of 3GES among patients enrolled in the CheckMate 8Y8 study (NCT03873402), an ongoing phase 3b clinical trial which evaluates the efficacy of nivolumab plus ipilimumab vs. nivolumab monotherapy in patients with previously untreated intermediate- or poor-risk aRCC.

Our study has two main limitations. First, it pivots on a *post*-*hoc* pooled analysis of those patients from the clinical trials CheckMate-009, CheckMate-010, and CheckMate-025 with enough clinical and molecular tumor data and those who consented to participate. Though Braun et al. have reported that these patients do not differ significantly with respect to survival from the whole population enrolled in the trials (9), this fact could lead to an uncontrolled selection bias. On a positive note, the availability of a control arm of patients treated with everolimus in the CheckMate-025 trial has allowed us to explore the predictive nature of our 3GES through the evaluation of biomarker–treatment interaction with the likelihood ratio test. The second limitation is the use of optimal cutoff thresholds to define our genes as high or low based on RNA-seq data. While promising, to confirm the utility of 3GES in a daily clinical practice scenario, our results should be validated with an easy-to-implement orthogonal technique such as RT-qPCR or nCounter assays. Furthermore, measuring immune cells from RNA expression data can be error-prone. Therefore, computational immune cell deconvolution results, although consistent, need to be interpreted cautiously in the absence of an independent validation method. On the other hand, though single-cell approaches such as single-cell sequencing or single-cell digital spatial profiling are still far from being applicable to daily clinical practice, they represent promising platforms to elevate precision oncology and biomarker development to the next level.

Today, either in daily clinical practice or in a clinical trial scenario, there are different treatment options available for the management of patients with accRCC. In this context, the development of tools to help in the decision-making process is mandatory. In this study, in addition to developing and validating the 3GES for predicting clinical benefit to the anti-PD-1 antibody nivolumab in the context of patients diagnosed with accRCC, we characterized its underlying clinical, molecular, and immune features. If the results of this study are definitively validated in other retrospective and large-scale, prospective studies, the 3GES will represent a valuable tool for guiding the design of ICB-based clinical trials in this scenario in the near future.

## Data availability statement

Publicly available datasets were analyzed in this study. All clinical, molecular, and immune tumor data from CheckMate-009, CheckMate-010, and CheckMate-025 trials used for this retrospective study have been made freely available through a **Supplementary Material** by Braun et al. (https://www.nature.com/articles/s41591-020-0839-y).

## Ethics statement

This study was approved by the Research Ethics Committee of Santiago-Lugo (2022/063) and conducted in accordance with the guidelines for Good Clinical Practice and the Declaration of Helsinki. Because this study is based on the analysis of publicly available anonymized data, informed consent was waived. Statements confirming compliance with ethical regulations, the committees that approved the protocol of CheckMate studies, and confirmation of informed consent from all study participants are included in the previous publications describing these trials (NCT01358721, NCT01354431, and NCT01668784).

## Author contributions

YZB: Data curation, Formal analysis, Investigation, Methodology, Writing – original draft, Writing – review & editing, Visualization. MF-P: Formal analysis, Writing – review & editing. UA-H: Writing – review & editing. MF-L: Writing – review & editing. LL-M: Writing – review & editing. SMG-A: Writing – review & editing. VV-R: Writing – review & editing. BG-P: Writing – review & editing. VC-L: Writing – review & editing. RV-V: Writing – review & editing. HL-R: Writing – review & editing. MK: Writing – review & editing. NF-D: Writing – review & editing. IG-T: Writing – review & editing. CF-B: Writing – review & editing. JG-G: Writing – review & editing. JMCT: Writing – review & editing. RL-L: Writing – review & editing, Resources, Supervision. JR-B: Resources, Supervision, Writing – review & editing, Conceptualization, Data curation, Formal analysis, Funding acquisition, Investigation, Methodology, Writing – original draft.
