# Supplementary material for: A three-gene expression score for predicting clinical benefit to anti-PD-1 blockade in advanced renal cell carcinoma
Source: Front Immunol. 2024 Apr 10;15:1374728. doi: 10.3389/fimmu.2024.1374728 (PMC11039903; doi:10.3389/fimmu.2024.1374728)

A three-gene expression score for predicting clinical benefit to anti-PD-1 blockade in advanced renal cell carcinoma

Yoel Z. Betancor[1,2], Miriam Ferreiro-Pantín[1,*], Urbano Anido-Herranz[1,3,*], Mar Fuentes-Losada[1,3], Luis León-Mateos[1,3,4], Silvia Margarita García-Acuña[5], Vanessa Vaamonde-Rodríguez[3], Beatriz García-Pinel[2,6], Víctor Cebey-López[1,3], Rosa Villaverde-Viaño[3], Helena Lombardía-Rodríguez[1], Martin Kotrulev[2,6], Natalia Fernández-Díaz[1,3], Iria Gomez-Tourino[2,6], Carlos Fernández-Baltar[7], Jorge García-González[1,3,4], Jose M. C. Tubio[2], Rafael López-López[1,3,4,8], Juan Ruiz-Bañobre[1,3,4,8,#]

Affiliations:

1 Translational Medical Oncology Group (ONCOMET), Health Research Institute of Santiago de Compostela (IDIS), University Clinical Hospital of Santiago de Compostela, University of Santiago de Compostela (USC), 15706 Santiago de Compostela, Spain.

2 Centre for Research in Molecular Medicine and Chronic Diseases (CiMUS), University of Santiago de Compostela (USC), 15706 Santiago de Compostela, Spain.

3 Department of Medical Oncology, University Clinical Hospital of Santiago de Compostela (SERGAS), University of Santiago de Compostela (USC), 15706 Santiago de Compostela, Spain.

4 Centro de Investigación Biomédica en Red de Cáncer (CIBERONC), Instituto de Salud Carlos III, 28029 Madrid, Spain.

5 Department of Pathology, University Clinical Hospital of Santiago de Compostela, University of Santiago de Compostela (USC), 15706 Santiago de Compostela, Spain.

6 Health Research Institute of Santiago de Compostela (IDIS), 15706 Santiago de Compostela, Spain.

7 Department of Urology, Complexo Hospitalario Universitario de Pontevedra, 36071, Pontevedra, Spain.

8 Co-senior authors.

* These authors contributed equally to this work.

[#]Corresponding author:

Dr. Juan Ruiz-Bañobre; Medical Oncology Department, University Clinical Hospital of Santiago de Compostela, Travesía da Choupana S/N, 15706 Santiago de Compostela, Spain. Phone: (034)981951470. ORCID: 0000-0003-0755-4295. Email: juan.ruiz.banobre@sergas.es

Supplementary Material

Supplementary Tables:

- Supplementary Table 1
- Supplementary Table 2
- Supplementary Table 3
- Supplementary Table 4
- Supplementary Table 5
- Supplementary Table 6
- Supplementary Table 7

Supplementary Figures:

- Supplementary Figure 1

Supplementary Table 1. Univariable Cox regression analyses for overall survival considering baseline clinicopathological characteristics among nivolumab treated patients included in the pooled cohort of the CheckMate-009, -010, and -025 trials.

**Characteristic**[a]	**HR (95% CI)**	***P***
Sex (male vs female)	1.69 (1.09 - 2.63)	0.019
Age (> vs ≤ median)	0.82 (0.57 - 1.18)	0.288
MSKCC risk (favorable vs unfavorable)	0.44 (0.29 - 0.68)	< 0.001
Sarcomatoid or rhabdoid (yes vs no)	1.73 (1.03 - 2.89)	0.039
Number of prior therapies (continuous)	1.23 (0.87 - 1.45)	0.360

[a]Baseline variables IMDC risk and site of metastases were excluded from univariable Cox regression analyses because these data were not available in most cases.

Abbreviations: CI, confidence interval; HR, hazard ratio; MSKCC, Memorial Sloan Kettering Cancer Center.

Supplementary Table 2. Univariable Cox regression analyses for overall survival among nivolumab treated patients included in the pooled cohort of the CheckMate-009, -010, and -025 trials.

**Characteristic**	**HR (95% CI)**	**FDR-adjusted *P***
SPOCD1 (high *vs* low)	3.14 (2.11 - 4.67)	< 0.001
MYO9B (high *vs* low)	4.17 (2.49 - 6.98)	0.003
AUP1 (high *vs* low)	2.72 (1.90 - 3.91)	0.003
HMGA1 (high *vs* low)	2.73 (1.89 - 3.95)	0.004
HIST2H2BF (high *vs* low)	3.27 (2.11 - 5.07)	0.006
USF1 (high *vs* low)	3.15 (2.04 - 4.85)	0.009
NUP62 (high *vs* low)	2.59 (1.80 - 3.71)	0.010
ARHGAP42 (high *vs* low)	0.39 (0.27 - 0.56)	0.017
ADCY5 (high *vs* low)	0.38 (0.26 - 0.56)	0.017
MOCS1 (high *vs* low)	0.36 (0.25 - 0.54)	0.022
RP5_1107A17.2 (high *vs* low)	2.95 (1.93 - 4.51)	0.025
PSMD10P2 (high *vs* low)	3.56 (2.16 - 5.87)	0.026
CLIC4 (high *vs* low)	0.30 (0.18 - 0.48)	0.035
CDH5 (high *vs* low)	0.37 (0.25 - 0.55)	0.039
HMMR (high *vs* low)	2.85 (1.87 - 4.34)	0.044
HSF2BP (high *vs* low)	2.50 (1.73 - 3.61)	0.048
DIAPH3 (high *vs* low)	2.75 (1.83 - 4.14)	0.049

Abbreviations: CI, confidence interval; FDR, False Discovery Rate; HR, hazard ratio.

Supplementary Table 3. Univariable logistic regression analyses for disease control and response among nivolumab treated patients included in the pooled cohort of the CheckMate-009, -010, and -025 trials.

	**Disease control**		**Response**	
**Characteristic**	**OR (95% CI)**	***P***	**OR (95% CI)**	***P***
MYO9B (high *vs* low)	0.28 (0.10 - 0.77)	0.014	0.37 (0.08 - 1.68)	0.199
AUP1 (high *vs* low)	0.45 (0.25 - 0.84)	0.011	0.57 (0.26 - 1.23)	0.153
HMGA1 (high *vs* low)	0.57 (0.30 - 1.07)	0.080	0.25 (0.09 - 0.69)	0.007
USF1 (high *vs* low)	0.29 (0.13 - 0.66)	0.003	0.21 (0.05 - 0.93)	0.039
NUP62 (high *vs* low)	0.48 (0.26 - 0.89)	0.019	0.53 (0.24 - 1.17)	0.116
ARHGAP42 (high *vs* low)	2.17 (1.15 - 4.09)	0.016	2.17 (0.93 - 5.08)	0.074
MOCS1 (high *vs* low)	2.36 (1.18 - 4.72)	0.015	1.61 (0.66 - 3.96)	0.299

Abbreviations: CI, confidence interval; OR, odds ratio.

Supplementary Table 4. Multivariable Cox regression analyses for overall survival among nivolumab treated patients from the pooled cohort of the CheckMate-009, -010, and -025 trials.

**Characteristic**	**HR (95% CI)**	***P***
MYO9b (high *vs* low)	1.43 (0.72 - 2.88)	0.310
AUP1 (high *vs* low)	1.40 (0.91 - 2.17)	0.127
HMGA1 (high *vs* low)	1.60 (1.05 - 2.46)	0.031
USF1 (high *vs* low)	1.51 (0.85 - 2.65)	0.157
NUP62 (high *vs* low)	1.74 (1.17 - 2.60)	0.007
ARHGAP42 (low *vs* high)	1.74 (1.15 - 2.63)	0.008
MOCS1 (high *vs* low)	1.58 (1.00 - 2.52)	0.051

Abbreviations: CI, confidence interval; HR, hazard ratio.

Supplementary Table 5. Univariable and multivariable Cox regression analyses for overall survival comparing different genes expression signatures among nivolumab treated patients included in the pooled cohort of the CheckMate-009, -010, and -025 trials.

	**Univariable analysis**		**Multivariable analysis**	
**Characteristic**[a]	**HR (95% CI)**	***P***	**HR (95% CI)**	***P***
Angio (> median *vs* ≤ median)	0.67 (0.47 - 0.95)	0.026	0.87 (0.60 - 1.25)	0.4
Teff (> median *vs* ≤ median)	1.39 (0.97 - 1.99)	0.070		
Myeloid (> median *vs* ≤ median)	1.13 (0.79 - 1.61)	0.501		
JAVELIN (> median *vs* ≤ median)	1.41 (0.99 - 2.02)	0.059		
TIS (> median *vs* ≤ median)	1.16 (0.81 - 1.65)	0.417		
3GES (favorable *vs* unfavorable)	0.32 (0.21 - 0.48)	< 0.001	0.33 (0.22 - 0.50)	< 0.001

Abbreviations: Angio, angiogenesis score; CI, confidence interval; HR, hazard ratio; JAVELIN, JAVELIN immune infiltration score; Myeloid, Myeloid cell infiltration score; Teff, T effector cell infiltration score; TIS, Tumor Inflammation Score.

Supplementary Table 6. Univariable and multivariable Cox regression analyses for overall survival among patients included in the TCGA-KIRP and TCGA-KICH cohort.

**KIRP** (n = 286)	**Univariable analysis**		**Multivariable analysis**	
**Characteristic**[a]	**HR (95% CI)**	***P***	**HR (95% CI)**	***P***
HMGA1 (high *vs* low)	3.48 (1.88 - 6.44)	<0.001	2.71 (1.37 - 5.38)	0.004
NUP62 (high *vs* low)	2.93 (1.56 - 5.51)	<0.001	2.38 (1.18 - 4.81)	0.015
ARHGAP42 (low *vs* high)	2.92 (0.90 - 9.45)	0.07	4.82 (1.47 - 15.81)	0.009
**KICH** (n = 64)				
HMGA1 (high *vs* low)	8.84 (1.10 - 70.7)	0.04	5.89 (0.71 - 49.00)	0.101
NUP62 (high *vs* low)	4.99 (1.24 - 20.02)	0.02	3.29 (0.78 - 13.69)	0.102
ARHGAP42 (low *vs* high)	0.23 (0.06 - 0.84)	0.03	0.37 (0.09 - 1.45)	0.153

Abbreviations: Abbreviations: TCGA-KIRP, The Cancer Genome Atlas-Kidney Renal Papillary Cell Carcinoma; TCGA-KICH, The Cancer Genome Atlas-Kidney Chromophobe.

Supplementary Table 7. Baseline patient and disease characteristics of 311 patients included in the pooled cohort of the CheckMate-009, -010, and -025 trials.

**Characteristic**	**Total** (n = 311)	**Favorable 3GES** (n = 123, 40%)	**Unfavorable 3GES** (n = 188, 60%)	***P***
**Clinical trial (%)**				0.913
CM-009 and -010	61	25 (20)	36 (19)	
CM-025	250	98 (80)	152 (81)	
**Age (%)**				0.051
Age > 63 years (median)	141	65 (53)	76 (41)	
Age ≤ 63 years	168	58 (47)	110 (59)	
NA	2			
**Sex (%)**				0.778
Female	82	34 (28)	48 (26)	
Male	229	89 (72)	140 (74)	
**MSKCC risk (%)**				0.039
Favorable	100	48 (41)	52 (29)	
Unfavorable	195	68 (59)	127 (71)	
NA	16			
**Sarcomatoid and/or rhabdoid differentiation (%)**				0.042
No	247	103 (92)	144 (83)	
Yes	39	9 (8)	30 (17)	
NA	25			
**Ploidy (%)**				1
Ploidy > 1.98 (median)	91	37 (46)	54 (45)	
Ploidy ≤ 1.98	108	43 (54)	65 (54)	
NA	112			
**Immunophenotype (%)**				0.290
Desert or excluded	24	13 (30)	11 (19)	
Infiltrated	79	31 (70)	48 (81)	
NA	208			
**wGII (%)**				0.035
wGII > 0.11 (median)	99	32 (40)	67 (56)	
wGII ≤ 0.11	100	48 (60)	52 (44)	
NA	112			
**ITH (%)**				0.053
ITH > 0.26 (median)	99	47 (59)	52 (44)	
ITH ≤ 0.26	100	33 (41)	67 (56)	
NA	112			

P values were computed excluding NA cases.

Abbreviations: CM, CheckMate; MSKCC, Memorial Sloan Kettering Cancer Center; ITH, Intra-Tumor Heterogeneity; NA, not available; wGII, weighted Genome Instability Index.

Supplementary Figure 1. Time-dependent ROC curve analyses for overall survival for 3GES among **(A)** nivolumab treated patients included in the pooled cohort of the CheckMate-009, -010, and -025 trials, and **(B)** patients included in the TCGA-KIRC cohort. Dark grey areas represent 95% confidence intervals of area under the curve (AUC).

A

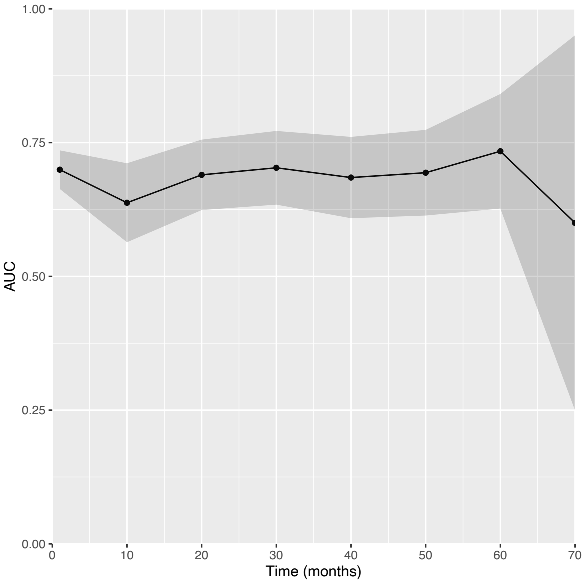

B

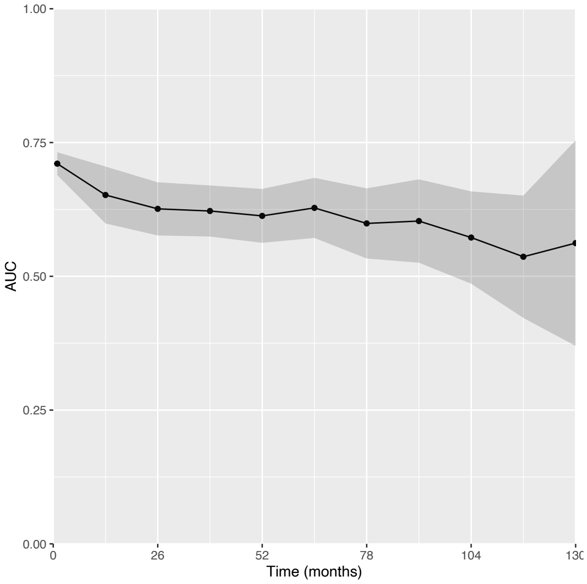

Supplement: Supplementary file 1 [file DataSheet_1.pdf]
